# Medikamentöse Tumortherapie von Kopf-Hals-Tumoren: zwischen Standardisierung und Personalisierung

**DOI:** 10.1007/s00106-025-01591-7

**Published:** 2025-04-22

**Authors:** Simon Laban

**Affiliations:** 1 Klinik für Hals-Nasen-Ohrenheilkunde und Kopf-Halschirurgie, Universitätsklinik Ulm, Ulm, Deutschland; 2 Kopf-Hals-Tumorzentrum des Comprehensive Cancer Center Ulm, Frauensteige 12, 89070 Ulm, Deutschland

**Keywords:** Immuncheckpointinhibitoren, Immuntherapie, Chemoradiotherapie, Neoadjuvante Systemtherapie, Personalisierte Medizin, Immune checkpoint inhibitors, Immunotherapy, Chemoradiotherapy, Neoadjuvant systemic therapy, Personalized medicine

## Abstract

In der medikamentösen Tumortherapie von Kopf-Hals-Malignomen gab es insbesondere in der palliativen Therapie neue Zulassungen, die zur Änderung von Therapiestandards führten. Gleichzeitig bestehen für einige Therapien molekulare Biomarker, die neben klinischen Faktoren zur Therapieauswahl in Betracht gezogen werden können. Diese Entwicklungen werden in der vorliegenden Arbeit zusammengefasst und mögliche Strategien zur Lösung der Spannung zwischen Standardisierung und Personalisierung aufgezeigt. Zusätzlich werden Ergebnisse der randomisierten Phase-III-Studien mit therapeutischen Antikörpern gegen die PD1/PD-L1-Achse in Kombination mit definitiver Chemoradiotherapie beschrieben und eingeordnet. Zum Schluss werden vor dem Hintergrund der Pressemitteilung über positive Ergebnisse der neoadjuvanten Studie KEYNOTE-689 für die lokoregionär fortgeschrittene, resektable Erkrankung mögliche Maßnahmen und Hinweise zur Integration neoadjuvanter Immuntherapien in den klinischen Alltag diskutiert.

**Zusatzmaterial online:**

Die Online-Version dieses Artikels (10.1007/s00106-025-01591-7) enthält eine zusätzliche Tabelle.

## Abstract (en)

In the field of systemic cancer therapy for head and neck neoplasms, new approvals have led to several changes in treatment standards, primarily in the palliative situation. At the same time, molecular biomarkers have been established that can contribute to therapeutic decision-making in addition to clinical factors. These developments are summarized, and potential strategies to resolve the tension between standardization and personalization are described. Results from randomized phase III studies with therapeutic antibodies targeting the PD1/PD-L1 axis in combination with definitive chemoradiation are described and discussed. Finally, inspired by the press release about the positive results of the neoadjuvant KEYNOTE-689 trial in locoregionally advanced resectable disease, potential measures and suggestions for integrating neoadjuvant immunotherapy into clinical routine are discussed.

Der vorliegende Artikel knüpft an das Referat von Prof. Dr. T.K. Hoffmann zur Systemtherapie bei Kopf-Hals-Malignomen aus dem Jahr 2012 an [21]. Darin wurden u. a. die Wirkmechanismen und Anwendungsgebiete traditioneller Chemotherapeutika beschrieben. Diese sollen hier nicht wiederholt werden, stattdessen wird auf das damalige Referat verwiesen.

## Bemühungen zur Standardisierung in der medikamentösen Tumortherapie

Das Konzept der evidenzbasierten Medizin (EBM) sorgte ab 1992 für einen Paradigmenwechsel in der Medizin [47]. Die EBM etablierte Ergebnisse aus klinischer Forschung als Entscheidungskriterium für die Bewertung von medizinischen Behandlungen und Diagnostik. Diese Bewegung führte zur Entwicklung evidenzbasierter Leitlinien, die auf Forschungsdaten beruhen und das Evidenzniveau dieser verfügbaren Forschungsergebnisse berücksichtigen. Randomisierte klinische Studien und deren Auswertung in Metaanalysen entwickelten sich zum Goldstandard für die Leitlinienentwicklung. Im Leitlinienprogramm Onkologie der Deutschen Krebsgesellschaft (DKG), Deutschen Krebshilfe (DKH) und Arbeitsgemeinschaft der Wissenschaftlichen Medizinischen Fachgesellschaften (AWMF) existieren aktuell 3 S3-Leitlinien (Mundhöhlenkarzinom [3], Larynxkarzinom [4], Oro- und Hypopharynxkarzinom [5]) und 2 in Erstellung/Überarbeitung befindliche S2k-Leitlinien (Diagnostik und Therapie des Lippenkarzinoms, Diagnostik und Management von Vorläuferläsionen des oralen Plattenepithelkarzinoms) für die Behandlung von Kopf-Hals-Tumoren. Durch diese Leitlinienarbeit wird eine standardisierte Patientenbehandlung nach aktuellen Forschungsergebnissen ermöglicht.

## Personalisierte Medizin – eine Gegenbewegung zur Standardisierung?

Die Grundhypothese der personalisierten Medizin besteht in der Berücksichtigung individueller, molekularer Veränderungen, z. B. auf Ebene des Genoms, Transkriptoms, Proteoms oder Epigenoms, für die Entwicklung diagnostischer und therapeutischer Verfahren. Auf den ersten Blick besteht im Streben nach Personalisierung ein Gegensatz zur Standardisierung. Bei etwas differenzierterer Betrachtung ergibt sich auch eine andere Interpretationsweise. Die Personalisierung von Diagnostik und Therapie nach molekularen Faktoren kann auch als Standardisierung auf höherer Ebene betrachtet werden. Aus klinischen Studien, in denen Biomarker für die Patientenselektion oder -gruppierung eingesetzt werden, kann Evidenz für die Standardisierung der Behandlung von Patienten mit entsprechender Biomarkerkonstellation entstehen. Personalisierte Medizin im Einklang mit evidenzbasierter Medizin resultiert jedoch in komplexeren Anforderungen an Studiendesign, -durchführung und -auswertung.

In der vorliegenden Übersichtsarbeit wird zunächst ein Überblick über historische Meilensteine in der Systemtherapie für Kopf-Hals-Tumoren gegeben, dann der aktuelle Stand des Spannungsfelds aus Standardisierung und Personalisierung in der medikamentösen Tumortherapie von Kopf-Hals-Tumoren zusammengefasst, und schließlich werden auch mögliche zukünftige Entwicklungen vorgestellt.

Zentrale Informationen zu den dargestellten Studien sind in Tabelle S1 aufgeführt.

## Beginn der Ära der Standardisierung und Personalisierung

Cetuximab, ein monoklonaler Epidermal-Growth-Factor-Receptor(EGFR)-Antikörper, war die erste zielgerichtete und personalisierte Therapie, die eine Zulassung für die Behandlung von Plattenepithelkarzinomen im Kopf-Hals-Bereich (HNSCC) erhielt. Zuerst wurde Cetuximab in Kombination mit Radiotherapie (RT) für lokoregionär fortgeschrittene HNSCC (LA-HNSCC) zugelassen [34, 36], später auch in Kombination mit Platin (P) und 5‑Fluoruracil (F) für rezidivierte und metastasierte HNSCC (RM-HNSCC), die keine platinhaltige Therapie innerhalb der letzten 6 Monate vor der Progression erhalten hatten [52]. Gleichzeitig stellte die Zulassung von Cetuximab in Kombination mit PF einen neuen Standard in der palliativen Therapie von RM-HNSCC dar, denn vorher wurden Cis‑/Carboplatin oder Methotrexat als Monotherapie oder auch in Kombination von P mit F [9] in der palliativen Erstlinie eingesetzt.

Die Einführung einer zielgerichteten Substanz für die Therapie von RM-HNSCC setzte auch den Startschuss für eine bessere Standardisierung der medikamentösen Therapie von RM-HNSCC, während eine weitere Personalisierung auf Basis der EGFR-Expression zunächst nicht stattfand. Die Wirksamkeit von Cetuximab schien, möglicherweise vor dem Hintergrund der weit verbreiteten und starken Expression des EGFR [40], nicht abhängig von der EGFR-Expression zu sein [52].

## Beginn der Ära der Immuntherapie

### Platinrefraktäre Situation

Die erste Zulassung für Programmed-Death-1(PD1)-Antikörper erhielt Nivolumab in der platinrefraktären Situation nach der randomisierten Phase-III-Studie CheckMate-141 (Progression innerhalb von 6 Monaten nach kurativ intendierter Systemtherapie oder nach platinhaltiger, palliativer Erstlinientherapie) [11]. Das verbesserte Gesamtüberleben („overall survival“, OS), der primäre Endpunkt, der CheckMate-141 war unabhängig von der Proteinexpression des Liganden von PD1, „programmed death ligand 1“ (PD-L1), gemessen mit dem DAKO-Klon 28‑8 der Fa. DAKO, (Carpinteria/CA, USA) im Vergleich zur Standardtherapie (Prüferauswahl einer Monotherapie mit Docetaxel, Methotrexat oder Cetuximab; „investigator’s choice“, IC). In der randomisierten Phase-III-Studie KEYNOTE-040 mit dem PD1-Antikörper Pembrolizumab, die ebenfalls in der platinrefraktären Situation mit vergleichbaren Ein- und Ausschlusskriterien und vergleichbarem Standardarm mit IC durchgeführt wurde, wurde der primäre Endpunkt des verbesserten OS in der Gesamtkohorte nicht erreicht [12]. In einer geplanten Subgruppenanalyse profitierten nur Patienten mit einer PD-L1-Expression in $\geq$ 50 % der Tumorzellen (Tumor Proportion Score, TPS), gemessen mit dem DAKO-Klon 22C3. Dies war die zweite Zulassung einer Immuntherapie für die palliative Systemtherapie von HNSCC und erstmals verbunden mit einem personalisierten Biomarker. In der damals vorliegenden Situation der bereits länger bestehenden Zulassung von Nivolumab ohne Einschränkung bezüglich der PD-L1-Expression führte dies jedoch zu einem deutlich geringeren Einsatz von Pembrolizumab im Vergleich zu Nivolumab.

Ebenfalls in der platinrefraktären Situation wurde die randomisierte Phase-III-Studie EAGLE mit Durvalumab, einem therapeutischen PD-L1-Antikörper, als Monotherapie oder in Kombination mit Tremelimumab, einem Antikörper gegen „cytotoxic T lymphocyte associated protein 4“ (CTLA4) im Vergleich zu IC einer Monotherapie mit Docetaxel, Methotrexat, Cetuximab oder F durchgeführt. Diese Studie erreichte den Endpunkt eines verbesserten OS im Vergleich zum Standardarm nicht [38]. In dieser Studie wurde als diagnostischer Antikörper der Ventana-Klon SP263 (Fa. Roche Diagnostics, Basel, Schweiz) eingesetzt.

Trotz der nahezu identischen Ein- und Ausschlusskriterien sowie Vergleichsarme dieser 3 Phase-III-Studien waren die Studienergebnisse jedoch recht unterschiedlich. Die genauen Ursachen für diese unterschiedlichen Ergebnisse lassen sich rückblickend nicht zweifelsfrei klären. Es existieren jedoch verschiedene mögliche, ungeplante Einflussfaktoren („confounder“):

#### 1. Die Prüferauswahl der Standardtherapie.

**Abb. 1:**
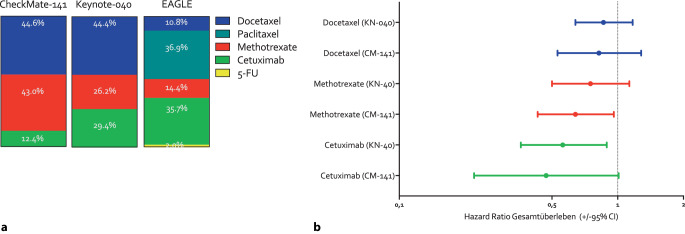
**a** Therapieauswahl im Standardarm („investigator’s choice“) in den Studien CheckMate-141 [11], KEYNOTE-040 [12] und EAGLE [38]. **b** Hazard Ratios für das Gesamtüberleben im Vergleich zum jeweiligen experimentellen Arm für Docetaxel, Methotrexat und Cetuximab in CheckMate-141 und KEYNOTE-040. *5‑FU* 5-Fluorouracil, *95%CI* 95%-Konfidenzintervall, *CM* CheckMate, *KN* KEYNOTE

Als Standardtherapie konnte vom Prüfer vor Randomisation aus Docetaxel, Methotrexat, Cetuximab (und in der EAGLE-Studie zusätzlich Paclitaxel, 5‑Fluoruracil, Capecitabin und Titansilicat 1 [TS-1]) ausgewählt werden, da in der platinrefraktären Situation kein einheitlicher Behandlungsstandard existierte. Die Wirksamkeit der Einzelsubstanzen in der Monotherapie scheint jedoch unterschiedlich zu sein, wie auch Subgruppenanalysen nahelegen [12, 38]. Die IC unterschied sich in den 3 Studien insbesondere im Anteil der Patienten, die Methotrexat oder Cetuximab erhielten. Zudem war Paclitaxel nur in der EAGLE-Studie verfügbar und schien etwas effektiver als Docetaxel zu sein (ungeplante Subgruppenanalyse; Hazard Ratio [HR] = 0,69; 95%-Konfidenzintervall [95%-KCI]: 0,44–1,13). Die Auswahl der Standardtherapie nach Studie ist in Abb. 1a und die Wirksamkeit der Substanzen Docetaxel, Methotrexat und Cetuximab im Vergleich zum jeweiligen experimentellen Arm in der CheckMate-141-Studie und der KEYNOTE-040-Studie in Abb. 1b zu sehen.

#### 2. Der Anteil von Patienten, die eine Immuntherapie erhielten.

Der Anteil von Patienten, die nach Progression im Standardarm eine Immuntherapie erhielten, war am geringsten in der CheckMate-141-Studie mit 10,1 %, gefolgt von 12,5 % in der KEYNOTE-040-Studie und 15,3 % in der EAGLE-Studie. Der Anteil immuntherapierter Patienten nach Progression im Rahmen der Studie stieg also, je später die Studien die Rekrutierung beendeten – aufgrund der Zulassungen von Nivolumab und Pembrolizumab und deren zunehmender Verfügbarkeit.

#### 3. Die Zusammensetzung der jeweiligen Studienpopulationen.

Diese unterschied sich u. a. geringfügig bezüglich der ethnischen Zugehörigkeit, der Primärtumorregion, des Geschlechts der Patienten sowie der Anteile an Patienten mit gutem Performance-Status der Eastern Cooperative Oncology Group (ECOG 0).

**Abb. 2:**
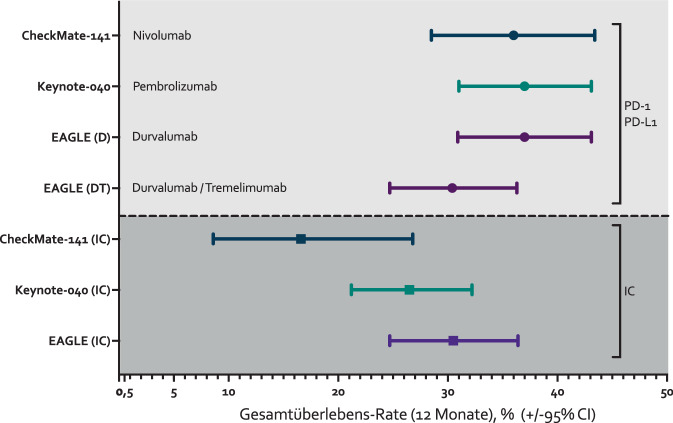
12-Monats-Gesamtüberlebensrate und 95%-Konfidenzintervall (*95%CI*) in den experimentellen Armen der Studien CheckMate-141 [11], KEYNOTE-040 [12] und EAGLE [38] sowie im jeweiligen Standardarm (*IC *„investigator’s choice“) bei platinrefraktären Patienten

In Summe führten diese (und möglicherweise weitere) Faktoren dazu, dass die Ergebnisse der 3 Studien sich insbesondere bezüglich der Standardarme deutlich unterschieden. Skizziert wird dies in einer Gegenüberstellung der 12-Monats-OS-Rate in % in Abb. 2. Während sich die Überlebensrate nach 12 Monaten in den experimentellen Armen inkl. der 95%-KI kaum unterschied, sind erhebliche Unterschiede in der 12-Monats-OS-Rate der jeweiligen Standardarme erkennbar.

### Palliative Erstlinientherapie

In der palliativen Erstlinientherapie für „platinsensible“ Patienten (Patienten mit Progress > 6 Monate nach platinhaltiger kurativer Therapie in der lokoregionär fortgeschrittenen Situation *und* bisher nicht in der rezidivierten oder metastasierten Situation behandelte Patienten) wurden 3 randomisierten Phase-III-Studien durchgeführt: Die KEYNOTE-048- [48], die CheckMate-651- [29] und die KESTREL-Studie [42]. Auch diese 3 Studien ähnelten sich bezüglich Ein‑, Ausschlusskriterien und Standardtherapie stark und hatten als Standardarm jeweils die Chemotherapiekombination aus der EXTREME-Studie [52] (Platin, 5‑Fluoruracil [PF] + Cetuximab). Im experimentellen Arm wurde in der KEYNOTE-048-Studie Pembrolizumab als Monotherapie oder in Kombination mit PF geprüft, während in der CheckMate-651-Studie Nivolumab + Ipilimumab und in der KESTREL-Studie Durvalumab als Monotherapie oder in Kombination mit Tremelimumab eingesetzt wurden. Es unterschieden sich die zunehmend komplexen statistischen Auswertungsstrategien und insbesondere die Ergebnisse der Studien erheblich [29, 42, 48].

Die KEYNOTE-048-Studie erreichte als einzige dieser 3 Studien ihren primären Endpunkt (Verbesserung des OS im Vergleich zum EXTREME-Regime) und führte zur Zulassung von Pembrolizumab und Pembrolizumab + PF in der palliativen Erstlinientherapie des HNSCC für Patienten mit einer PD-L1-Expression in Tumor- und/oder Immunzellen innerhalb des Tumors (Combined Positive Score; CPS ≥ 1; in den USA ist Pembrolizumab + PF unabhängig vom PD-L1-Status zugelassen). Es ergab sich für Pembrolizumab + PF und Pembrolizumab-Monotherapie ein verbessertes OS für die Subgruppe der Patienten mit CPS ≥ 1 und CPS ≥ 20. Für Pembrolizumab + PF wurde auch in der Gesamtpopulation ein verbessertes OS erreicht (HR 0,77; 95%-KI: 0,63–0,93; *p* = 0,0034). Für die Gesamtkohorte ohne Personalisierungsfaktor wurde für Pembrolizumab-Monotherapie zumindest eine Nichtunterlegenheit (Nichtunterlegenheitsgrenze: HR = 1,2; HR 0,85 95%-KI: 0,71–1,03; *p* = 0,0456) zum EXTREME-Kontrollarm erreicht [48].

Die CheckMate-651- und die KESTREL-Studie erreichten die primären Endpunkte nicht.

**Abb. 3:**
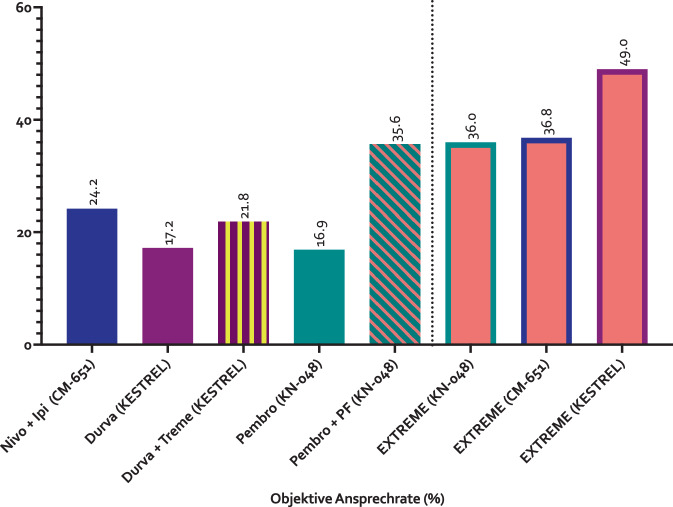
Objektive Ansprechrate („objective response rate“, ORR) in % in den experimentellen und Standardarmen der Studien KEYNOTE-048 (*KN*) [48], CheckMate-651 (*CM*) [29] und KESTREL [42]. *Durva* Durvalumab, *Ipi *Ipilimumab, *Nivo* Nivolumab, *Pembro* Pembrolizumab, *Treme* Tremelimumab

Betrachtet man zunächst die objektiven Ansprechraten („objective response rate“; ORR) der 3 Studien nach Response Evaluation Criteria In Solid Tumors, RECIST 1.1 (Abb. 3), fällt auf, dass die Ansprechraten im Standardarm der KEYNOTE-048- und CheckMate-651-Studie und die Kombination aus Immun- und Chemotherapie (Pembrolizumab + PF) vergleichbar mit der Ansprechrate in der Originalpublikation des EXTREME-Protokolls waren, während die Ansprechrate im Standardarm der KESTREL-Studie deutlich höher ausfiel. Bei den chemotherapiefreien experimentellen Armen war die ORR von Nivolumab + Ipilimumab am höchsten, gefolgt von Durvalumab + Tremelimumab, Durvalumab-Monotherapie und zuletzt Pembrolizumab-Monotherapie (Abb. 3). Die Betrachtung der Ansprechrate erklärt somit die Ergebnisse nicht.

**Abb. 4:**
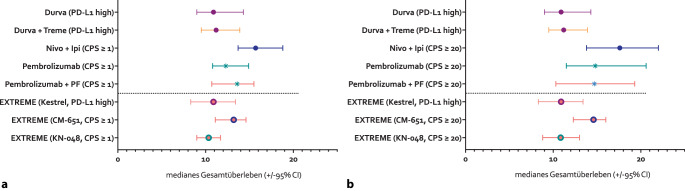
Medianes Gesamtüberleben ± 95%-Konfidenzintervall (*95%CI*) nach Behandlungsarm und PD-L1-Status in den Studien KEYNOTE-048 [48], CheckMate-651 [29] und KESTREL [42] für **a** Patienten mit Combined Positive Score (*CPS*) ≥ 1 und **b** CPS ≥ 20. In der KESTREL-Studie keine Subgruppenanalyse nach CPS, sondern nur Unterscheidung zwischen hohem PD-L1 (*PD-L1 high*) und niedrigem PD-L1 (*PD-L1 low*). Daher zum Vergleich Patienten mit *PD-L1 high* und *PD-L1 low *in beiden Grafiken dargestellt*. Durva* Durvalumab, *Ipi *Ipilimumab, *Nivo* Nivolumab, *Treme* Tremelimumab

Das mediane OS in den Standardarmen der KEYNOTE-048- und KESTREL-Studie war vergleichbar und ähnelte den Ergebnissen in der EXTREME-Studie [29, 48, 52]. Die CheckMate-651-Studie erreichte im Standardarm auffällig bessere Ergebnisse. Die experimentellen Arme der KESTREL-Studie blieben hinter den Ergebnissen der KEYNOTE-048- und der CheckMate-651-Studie zurück. Nominell wurden in der CheckMate-651-Studie die besten OS-Ergebnisse für den Standard- und experimentellen Arm erreicht. Innerhalb der geplanten statistischen Auswertungen wurde jedoch der primäre Endpunkt verfehlt. Zur besseren Einordnung des OS in der KEYNOTE-048-, der CheckMate-651- und der KESTREL-Studie ist das mediane OS mit 95%-KI in den Kohorten mit CPS ≥ 1 und CPS ≥ 20 für die jeweiligen experimentellen und Standardarme in Abb. 4 grafisch gegenübergestellt (**Cave**: In der KESTREL-Studie fand keine Subgruppenanalyse nach CPS statt, sondern es wurde zwischen hohem PD-L1 [PD-L1 high] und niedrigem PD-L1 [PD-L1 low] unterschieden; dabei wies die Gruppe PD-L1 high eine PD-L1-Expression in 50 % der Tumorzellen oder in 25 % der Immunzellen auf [35]).

## Subgruppenanalysen nach CPS in der Studie KEYNOTE-048

Zusätzlich zu den im Protokoll definierten Auswertungen wurden später Subgruppenanalysen nach CPS für Patienten mit CPS < 1, CPS = 1–19 und CPS ≥ 20 durchgeführt und publiziert [39]. Derartige Subgruppenanalysen sind in die Bewertung der European Medicines Agency für die Zulassung in Europa eingegangen und wurden auch in einem 2019 veröffentlichten Statement der Arbeitsgemeinschaft Internistische Onkologie (AIO) berücksichtigt. Bei diesen Subgruppenanalysen ist zu beachten, dass es sich mit Ausnahme der Analyse der Patienten mit CPS ≥ 20 um ungeplante, explorative Subgruppenanalysen handelt, für die die Studie nicht konzipiert war (Power, Fallzahlkalkulation).

In der Subgruppe mit CPS < 1 ergab sich für Pembrolizumab-Monotherapie (*n* = 44) kein signifikanter Vorteil im medianen Überleben im Vergleich mit EXTREME (*n* = 45). In der Subgruppe mit CPS = 1–19 wurden 124 Patienten dem Pembrolizumab-Monotherapie-Arm randomisiert zugeteilt und mit 133 Patienten im EXTREME-Arm verglichen. Das mediane OS war auch in dieser Subgruppe für Pembrolizumab-Monotherapie nicht signifikant besser als im EXTREME-Arm. Nur in der Subgruppe mit CPS ≥ 20 wurde für Pembrolizumab (*n* = 133) im Vergleich mit EXTREME (*n* = 122) ein signifikanter Überlebensvorteil beobachtet. Die Ergebnisse sind in Tab. 1 zusammengefasst.

**Tab. 1:** Medianes Gesamtüberleben in der KEYNOTE-048-Studie in einer ungeplanten Subgruppenanalyse nach Combined Positive Score (CPS) [39]

	CPS < 1				CPS = 1–19				CPS ≥ 20			
	Medianes OS	95%-KI	HR (95%-KI)	*p*-Wert	Medianes OS	95%-KI	HR (95%-KI)	*p*-Wert	Medianes OS	95%-KI	HR (95%-KI)	*p*-Wert
Pembrolizumab-Monotherapie	7,9	4,7–13,6	1,51 (0,96–2,37)	0,9624	10,8	9,0–12,6	0,86 (0,66–1,12)	0,1283	14,8	11,5–20,6	0,58 (0,44–0,78)	0,0001
EXTREME	11,3	9,1–15,9			10,1	8,7–12,1)			10,7	8,8–12,8		
Pembrolizumab + PF	11,3	9,5–14,0	1,21 (0,76–1,94)	0,7893	12,7	9,4–15,3	0,71 (0,54–0,94)	0,0073	14,7	10,3–19,3	0,60 (0,45–0,82)	0,0004
EXTREME	10,7	8,5–15,9			9,9	8,6–11,5			11,0	9,2–13,0		

*OS* Gesamtüberleben („overall survival“), *95%-KI* 95%-Konfidenzintervall, *HR* Hazard Ratio

Für Pembrolizumab + PF (*n* = 39) unterschied sich das mediane Überleben in der Subgruppe mit CPS < 1 ebenfalls nicht signifikant vom medianen Überleben im EXTREME-Arm (*n* = 43). Allerdings wurde für die Subgruppen mit CPS = 1–19 (*n* = 116) und CPS ≥ 20 für Pembrolizumab + PF (*n* = 126) im Vergleich mit EXTREME (CPS = 1–19: *n* = 125; CPS ≥ 20: *n* = 110) ein signifikant verlängertes medianes OS nachgewiesen. Auch diese Daten sind in Tab. 1 dargestellt.

In einer explorativen Post-hoc-Langzeitanalyse der KEYNOTE-048-Studie wurde mehrere Jahre nach der finalen Analyse für die protokollgemäß definierten Subgruppen eine erneute Auswertung des OS, des progressionsfreien Überlebens („progression-free survival“, PFS) und des PFS2 (PFS der Folgetherapie) mit einem Follow-up von etwa 4 Jahren durchgeführt [33].

In dieser Auswertung konnten die Ergebnisse der finalen Analyse von 2019 bestätigt werden. Das mediane OS ist in den Subgruppen mit CPS ≥ 1 und CPS ≥ 20 auch mit längerem Follow-up signifikant länger für die Patienten, die mit Pembrolizumab-Monotherapie oder Pembrolizumab + PF behandelt wurden. Auch für die Gesamtkohorte wurde mit längerem Follow-up eine signifikante Verbesserung des OS beobachtet. Diese bestand wie bereits beschrieben zum Zeitpunkt der finalen Auswertung nicht. Die Ergebnisse sind in Tab. 2 dargestellt.

**Tab. 2:** Medianes Gesamtüberleben in der KEYNOTE-048-Studie nach 4 Jahren Follow-up abhängig vom Combined Positive Score (CPS) [33]

	Gesamtkohorte				CPS ≥ 1				CPS ≥ 20			
	Medianes OS	95%-KI	HR (95%-KI)	*p*-Wert	Medianes OS	95%-KI	HR (95%-KI)	*p*-Wert	Medianes OS	95%-KI	HR (95%-KI)	*p*-Wert
Pembrolizumab-Monotherapie	11,5	10,3–13,5	0,81 (0,68–0,97)	0,00994	12,3	10,8–14,8	0,74 (0,61–0,89)	0,0008	14,9	11,5–20,6	0,61 (0,46–0,81)	0,00034
EXTREME	10,7	9,3–12,1			10,4	9,0–11,7			10,8	8,8–12,8		
Pembrolizumab + PF	13,0	10,9–14,7	0,71 (0,59–0,85)	0,00008	13,6	10,7–15,5	0,64 (0,53–0,78)	0,00001	14,7	10,3–19,3	0,62 (0,46–0,84)	0,00082
EXTREME	10,7	9,3–11,7			10,6	9,1–11,7			11,1	9,2–13,0		

*OS* Gesamtüberleben („overall survival“), *95%-KI* 95%-Konfidenzintervall, *HR* Hazard Ratio

Bemerkenswert ist ein plateauartiger Verlauf der Kaplan-Meier-Kurve bei etwa 30 % für CPS ≥ 20 der Patienten, die mit Pembrolizumab-Monotherapie oder Pembrolizumab + PF behandelt wurden, und bei etwa 20 % für Patienten mit CPS ≥ 1 und der Gesamtkohorte. Nach 4 Jahren Follow-up waren 21,6 % der Patienten im Pembrolizumab-Monotherapie-Arm mit CPS ≥ 20; 16,7 % mit CPS ≥ 1 und 15,4 % der Gesamtkohorte am Leben, im Vergleich zu 8,0 %; 5,9 % und 6,6 % der Gesamtkohorte. Im Pembrolizumab + PF-Arm lebten nach 4 Jahren noch 28,6 % mit CPS ≥ 20; 21,8 % mit CPS ≥ 1 und 19,4 % der Gesamtkohorte. Es scheint sich langfristig ein geringer Vorteil für Patienten, die mit Pembrolizumab + PF im Vergleich zu Pembrolizumab-Monotherapie behandelt wurden, abzuzeichnen.

Dieser Überlebensunterschied scheint sich nach 5 Jahren jedoch weiter zu relativieren [20]. Die 5‑Jahres-Überlebensrate lag in der Kohorte mit Pembrolizumab-Monotherapie mit CPS ≥ 20 bei 19,9 %; mit CPS ≥ 1 bei 15,4 % und in der Gesamtkohorte bei 14,4 %. Im Pembrolizumab + PF-Arm lag die 5‑Jahres-Überlebensrate von Patienten mit CPS ≥ 20 bei 23,9 %; mit CPS ≥ 1 bei 18,2 % und in der Gesamtkohorte bei 16,0 %.

Was die Dauer des Ansprechens auf die Therapie betrifft, ergibt sich ein anderes Bild. Nach Pembrolizumab-Monotherapie lag die Dauer des Ansprechens für Patienten mit CPS ≥ 20 bei 23,4 Monaten (95%-KI: 6,9–38,7); für Patienten mit CPS ≥ 1 bei 24,8 Monaten (95%-KI: 6,9–35,9) und nach Pembrolizumab + PF bei 7,0 (95%-KI: 4,1–21,9); 6,7 (95%-KI: 3,9–14,1) und 6,7 (3,9–13,2) Monaten. Das deutlich kürzere mediane Ansprechen bei der Kombination mit Chemotherapie resultiert am ehesten aus dem hohen Anteil an Patienten, die nur auf die Chemotherapiekomponente ansprachen und keine lang andauernde immunvermittelte Tumorkontrolle erreichten.

### Implikationen für die Personalisierung nach CPS in der Erstlinientherapie

Vorangestellt sei die Anregung, dass zur weiteren Entwicklung der Behandlungsoptionen und Evidenzlage der Einschluss in klinische Interventionsstudien mit neuen Therapieansätzen (Phase I–IV) in der palliativen Systemtherapie stets in Erwägung gezogen werden sollte und Patienten, falls verfügbar und gewünscht, ein derartiges Studienangebot gemacht werden sollte. Klinische Studien sind in der heutigen Zeit eine sinnvolle und attraktive Option für Patienten, am medizinischen Fortschritt teilzuhaben und auch selbst einen Beitrag dazu zu leisten.

In der palliativen Erstlinie sollte analog zu den Zulassungsstudien zwischen platinrefraktären (Progress < 6 Monate nach platinhaltiger kurativer Therapie für lokoregionär fortgeschrittene Erkrankung) und platinnaiven Patienten unterschieden werden. Hier beziehe ich mich zunächst auf Patienten, die bisher keine platinhaltige Therapie erhalten haben bzw. keinen Progress < 6 Monate nach platinhaltiger kurativer Therapie aufwiesen. Die genannten Ergebnisse der KEYNOTE-048- und weiterer Studien lassen einige klare Personalisierungsempfehlungen, aber auch einigen Interpretationsspielraum zu. Die detaillierte Kenntnis der Studienergebnisse, der Patientenfaktoren und praktische Erfahrungen mit unterschiedlichen Therapien verringern die Spannung zwischen Personalisierung und Standardisierung und ermöglichen eine patientenangepasste Therapieauswahl. Zu diesem Zweck ist auch eine koordinierte Weiterbildung in der medikamentösen Tumortherapie, z. B. durch Erwerb der Zusatzbezeichnung „Medikamentöse Tumortherapie“, ratsam und eine kontinuierliche Aktualisierung der Kenntnisse erforderlich. Durch eine derartige Spezialisierung zentralisierter Versorgung in Kopf-Hals-Tumorzentren können die Behandlungsergebnisse von Patienten erheblich verbessert werden, wie die Studie „Wirksamkeit der Versorgung in onkologischen Zentren“ (WiZen) eindrucksvoll nachgewiesen hat [19].

#### Erstlinientherapie für Patienten mit CPS < 1

Aufgrund der Zulassungssituation ist klar, dass in der Erstlinie für Patienten mit CPS < 1, die noch keine platinhaltige Therapie erhalten haben, nur eine platinbasierte Chemotherapie, üblicherweise kombiniert mit Cetuximab, infrage kommt. Hier stellt das EXTREME-Schema [52] in erster Linie den Standard dar. Eine Alternative ist das TPEx-Schema [26], in dem die Cisplatindosis auf 75 mg/m^2^ reduziert wurde, F durch Docetaxel 75 mg/m^2^ (T) ersetzt wurde und nur 4 statt 6 Zyklen verabreicht werden, jedoch obligat mit Febrile-Neutropenie-Prophylaxe mit granulozytenstimulierendem Faktor (G-CSF). Die Cetuximab-Gabe erfolgt analog zum EXTREME-Schema. An sich war die TPEx-Studie eine negative Phase-II-Studie, denn die Statistik war ausgelegt, eine Überlegenheit von TPEx gegenüber EXTREME für das OS mit einer HR von 0,72 zu zeigen. Auf Nichtunterlegenheit wurde nicht geprüft. Der primäre Endpunkt wurde nicht erreicht, dennoch hat sich das TPEx-Protokoll in den Augen vieler Behandler als Alternative etabliert. Die Ansprechraten (jeweils 57 %) und das mediane OS (14,5; 95%-KI: 12,5–15,7; vs. 13,4 Monate; 95%-KI: 12,2–15,4) waren vergleichbar, während die Rate an unerwünschten Arzneimittelwirkungen („adverse events“, AE) im TPEx-Arm mit 12 % geringer als im EXTREME-Arm (22 %) war. Die geringere Rate an AE ist einerseits der geringeren Anzahl von geplanten Kombinationstherapiezyklen (4 vs. 6), der G‑CSF-Prophylaxe für alle Patienten im TPEx-Arm und möglicherweise auch dem Austausch von F gegen T geschuldet.

Eine weitere Alternative stellt die Kombination aus Carboplatin, Paclitaxel und Cetuximab (PCC) wöchentlich oder 3‑wöchentlich dar [28, 46], für die in der Phase-II-CETMET-Studie auch eine Nichtunterlegenheit gezeigt wurde [51]. Auch für diese Kombination sind die Ansprechrate und die Überlebensergebnisse vergleichbar mit dem EXTREME-Schema. Ein Vorteil dieser Kombination ist die ambulante Applikationsmöglichkeit aufgrund der geringeren Notwendigkeit einer intensiven Flüssigkeitsregimes durch den Ersatz von Cisplatin durch Carboplatin. Allerdings ist der Ersatz von Cis- durch Carboplatin auch beim EXTREME- und TPEx-Schema vorgesehen und möglich.

Einschränkend muss man sagen, dass, abgesehen vom EXTREME-Arm der KEYNOTE-048-Studie, in keiner der Studien mit platinbasierter Therapie eine Subgruppenanalyse nach CPS durchgeführt wurde und daher keine Aussage zur Performance dieser alternativen Regime in den Subgruppen nach CPS gemacht werden kann.

#### Erstlinientherapie für Patienten mit CPS = 1–19

Patienten mit CPS ≥ 1 sind grundsätzlich Kandidaten für eine Therapie mit Pembrolizumab, sofern keine Kontraindikationen vorliegen (z. B. therapiebedürftige Autoimmunerkrankungen, die eine systemische Steroidtherapie erfordern). Für Patienten mit CPS = 1–19 kann zwischen Pembrolizumab-Monotherapie oder Pembrolizumab + PF gewählt werden. In der KEYNOTE-048-Studie wurden die beiden experimentellen Arme formell nicht miteinander verglichen, und die Studie war dafür nicht gepowert. Die Entscheidung, ob eine Kombination mit Chemotherapie oder die Monotherapie eingesetzt wird, obliegt dem Behandler. Die Kombinationstherapie führt – wie bereits beschrieben – zu einer höheren Ansprechrate und weniger Progressionen während der ersten 6 Monate der Therapie. Die Langzeitdaten der KEYNOTE-048-Studie legen auch ein etwas höheres Langzeitüberleben für Patienten nahe, die mit einer Kombinationstherapie behandelt wurden, wobei der Vergleich zwischen Pembrolizumab und Pembrolizumab + PF formal nicht untersucht wurde. Andererseits führt die Hinzunahme der Chemotherapie zu einer Steigerung der AE-Rate im Vergleich zur Monotherapie mit Pembrolizumab (55 % vs. 85 % AE ≥ Grad 3). Ein weiterer Vorteil der Monotherapie besteht darin, dass im Therapieverlauf schnell erkennbar wird, ob ein immunvermitteltes Ansprechen, das ein Hinweis auf eine langfristige Tumorkontrolle ist, erreicht wird. Die Chemotherapie verschleiert, ob das Ansprechen immunvermittelt ist. Wählt man die Monotherapie, sollte ein frühes Restaging möglichst nach 2–3 Zyklen und eine engmaschige Evaluation auf klinische Zeichen eines Progresses erfolgen.

Als zusätzliche Entscheidungsfaktoren spielen die Tumorsymptomlast, die Wachstumsdynamik (falls bekannt) sowie Allgemeinzustand und Vorerkrankungen des Patienten eine Rolle. Hohe Tumorsymptomlast und rasche Wachstumsdynamik, zusammen genommen auch oft als Remissionsdruck bezeichnet, sind Faktoren für die Kombination von Pembrolizumab und Chemotherapie, während ein reduzierter Allgemeinzustand oder relevante Vorerkrankungen eher für eine Monotherapie sprechen. In einer von der Arbeitsgemeinschaft Onkologie der Deutschen Gesellschaft für Hals-Nasen-Ohren-Heilkunde, Kopf-Hals-Chirurgie (DGHNO-KHC) durchgeführten Umfrage gaben etwa 2/3 der befragten für Patienten mit CPS = 1–19 als Präferenz die Kombinationstherapie an und 1/3 Pembrolizumab-Monotherapie [24].

#### Erstlinientherapie für Patienten mit CPS ≥ 20

Für Patienten mit CPS ≥ 20 kann ebenfalls zwischen Pembrolizumab-Monotherapie oder Pembrolizumab + PF gewählt werden. In dieser Subgruppe scheinen frühe Progressionen jedoch nicht häufiger unter Monotherapie als unter Kombinationstherapie vorzukommen. Die Inkaufnahme der relevant höheren Toxizität der Kombination erscheint nur bei Patienten mit hoher Tumorsymptomlast oder schneller Wachstumsdynamik erforderlich zu sein. Allerdings bleibt unklar, ob in dieser Subgruppe die Chemotherapie langfristig doch zu etwas höherem Überleben führt [33]. In der Umfrage der AG Onkologie der DGHNO-KHC war das Meinungsbild entsprechend umgekehrt. Etwa 2/3 der Behandler bevorzugen bei Patienten mit CPS ≥ 20 die Monotherapie und 1/3 die Kombinationstherapie [24].

### Palliative Zweitlinientherapie

Nach Progression auf eine Erstlinientherapie ist die Folgetherapie abhängig von der vorhergegangenen Therapie. Einschränkend bleibt zu bedenken, dass die Studienergebnisse für platinrefraktäre Patienten zuerst vorlagen und deren Ergebnisse nicht uneingeschränkt auf die nun veränderte, komplexere Situation in der Erstlinientherapie übertragbar sind.

Platinrefraktäre Patienten, also Patienten, die in der Erstlinie eine platinbasierte Therapie erhielten oder in der lokoregionär fortgeschrittenen Situation Platin erhalten haben, können mit Nivolumab oder Pembrolizumab (nur für TPS $\geq$ 50 zugelassen) behandelt werden. Seit der Zulassung von Pembrolizumab und Pembrolizumab + PF in der Erstlinientherapie betrifft sind dies in erster Linie Patienten, die einen CPS < 1 hatten. Eine Rebiopsie kann nach der ersten Progression erfolgen, um erneut die PD-L1-Expression zu bestimmen oder um eine weitere molekulare Diagnostik einzuleiten, falls es erneut zu einem Progress kommt. Dies ist für Patienten sinnvoll, die in gutem Allgemein- und Gesundheitszustand sind und deren Tumoren mit vertretbarem Risiko biopsiert werden können.

Für Patienten die in der Erstlinie einen PD1-Antikörper als Monotherapie erhalten haben, wird üblicherweise eine platinhaltige Therapie eingesetzt, sofern die Patienten für eine Kombinationstherapie geeignet sind. Die Therapieschemata, die hier üblicherweise eingesetzt werden, sind EXTREME, TPEx oder PCC. Alternativ ist für Patienten mit eingeschränktem Performance-Status auch eine Monotherapie mit Cis- oder Carboplatin oder einem Taxan denkbar. Prospektive Daten liegen auch für diese Situation nur sehr eingeschränkt vor.

Für Patienten, die bereits Platin und PD1-Antikörper in der Erstlinie erhalten haben, liegen keine guten Daten vor. Wurde die Kombination aus Pembrolizumab + PF gegeben, könnte eine Monotherapie mit einem Taxan, Methotrexat oder Cetuximab erfolgen. Denkbar ist auch eine Kombination aus Taxan und Cetuximab.

In der Interlink-1-Studie wurde in dieser Platin- und PD1-refraktären Population eine Monotherapie mit Cetuximab + Placebo als Standardtherapie mit einer experimentellen Kombination aus Cetuximab und dem NKG2A-Antikörper Monalizumab verglichen [1]. Die Studie war jedoch negativ.

Wurde bereits eine platinhaltige Therapie und davor oder danach ein PD1-Antikörper gegeben, ist die Situation noch komplexer, da in dieser Situation möglicherweise bereits ein Taxan als Chemotherapiekombination eingesetzt wurde (z. B. TPEx oder PCC). Eine Standardtherapie gibt es hier nicht. Optionen bestehen im Einsatz von Methotrexat oder Gemcitabin als Monotherapie. Eine neue Option ist eine molekular basierte Therapie auf Basis des Sozialgesetzbuchs V nach Durchführung eines molekularen Tumorboards, z. B. an einem Zentrum für personalisierte Medizin, falls Mutationen, Fusionen oder überexprimierte, molekulare Targets identifiziert werden können und es Evidenz für eine mögliche Wirksamkeit von zielgerichteten Substanzen für diese molekularen Veränderungen gibt. Voraussetzung für die Bewilligung ist die Darlegung der Behandlungssituation des Patienten (u. a. keine etablierte Standardtherapie, zum Tod führende Erkrankung, keine verfügbare Studie) und die Rationale für eine Wirksamkeit auf Basis vorliegender Studienergebnisse in der vorliegenden Tumorentität aus frühen Studien der Phase I–II in einem Kostenübernahmeantrag an die Krankenkasse.

## Integration der Immuntherapie in die kurative Therapie

In den letzten Jahren sind mehrere große prospektive, randomisierte Phase-III-Studien in der kurativen Situation bei LA-HNSCC durchgeführt worden, in welche Antikörper der PD1/PD-L1-Achse zusätzlich zur Standardtherapie integriert wurden.

Zunächst wurden 3 große Phase-III-Studien für HNSCC-Patienten durchgeführt, die nichtchirurgisch durch eine definitive Chemoradiotherapie (CRT) behandelt werden sollten (nichtresektabel oder Kandidaten für Organerhalt): die Studien JAVELIN Head and Neck 100 [27], GORTEC-REACH [17, 30] und KEYNOTE-412 [43].

### Studie JAVELIN Head and Neck 100

Haupteinschlusskriterien waren Patienten mit neu diagnostiziertem HNSCC im Stadium III/IV (nach AJCC-Klassifikation, Version 7; Oropharynxkarzinom p16+: T4, N2c oder N3; Oropharynxkarzinom p16− und Karzinome in Mundhöhle, Pharynx oder Larynx im Stadium III/IVa oder IVb) mit gutem Performance-Status, d. h. ECOG 0/1, die für eine nichtchirurgische platinhaltige Therapie geeignet sind. In der doppelblinden placebokontrollierten Studie JAVELIN HN 100 wurden 697 Patienten randomisiert einem Studienarm zugeteilt [27]. Als Standardarm (*n* = 347) wurde ein Placebo mit einer definitiven CRT mit Hochdosis-Cisplatin (100 mg/m^2^, 3 × alle 3 Wochen) und 70 Gy in Standardfraktionierung kombiniert (analog zu dem Schema bei Forastiere et al. [49]). Im experimentellen Arm wurden *n* = 350 Patienten mit Avelumab (10 mg/kg, alle 2 Wochen, Beginn eine Woche vor CRT), einem PD-L1-Antikörper, und der genannten CRT behandelt. Nach Ende der CRT wurde Avelumab für bis zu einem Jahr adjuvant fortgeführt.

Es erfolgte eine 1:1-Block-Randomisation, stratifiziert nach Primärtumorstatus (< T4 vs. T4), Nodalstatus (N0, N1, N2a, N2b vs. N2c, N3) und p16-Status (Oropharynxkarzinome p16+ vs. Oropharynxkarzinome p16−, Nichtoropharynxkarzinome). Als primärer Endpunkt war eine Verbesserung des PFS (HR $\leq$0,68; α‑Fehler mit einseitiger Signifikanz von 0,025; Power = 90 %) vorgesehen. Im Rahmen einer hierarchischen Teststrategie war bei positivem primärem Endpunkt eine Testung für das OS vorgesehen (HR $\leq$0,75; α‑Fehler mit einseitiger Signifikanz von 0,025; Power = 80 %). Die PD-L1-Testung erfolgte mittels VENTANA-PD-L1-Assay (SP263) der Fa. Roche Diagnostics (Basel, Schweiz) und wurde bei der Stratifizierung nicht berücksichtigt.

Im Dezember 2019 erfolgte eine geplante Zwischenanalyse nach 224 Events (77,5 % der statistisch benötigten Events), die zu einem Studienabbruch im März 2021 führte. Der Grund für die Terminierung der Studie war ein Mangel an Effektivität bezüglich des PFS im experimentellen Arm (HR = 1,21;95%-KI: 0,93–1,57). Die experimentelle Therapie führte weder zu einer Verbesserung des PFS, OS oder der Ansprechrate, sondern sogar zu nominell geringgradig schlechteren Ergebnissen.

### Studie GORTEC-REACH

Die GORTEC-REACH-Studie war eine unverblindete, randomisierte Phase-III-Studie und bestand aus 2 Substudien. Eine Kohorte für platingeeignete Patienten (Kohorte 1) und eine für platinungeeignete Patienten (Kohorte 2). In beiden Kohorten wurde eine RT mit 70 Gy in 6,5 Wochen (Fraktionierung analog zu [34]) appliziert. Kohorte 1 erhielt im Standardarm eine Hochdosis-Chemotherapie mit 3 Zyklen Cisplatin 100 mg/m^2^ analog zu dem Schema bei Forastiere et al. [49], während im Standardarm von Kohorte 2 Cetuximab (400 mg/m^2^ „loading dose“, danach 250 mg/m^2^ wöchentlich; insgesamt 7 Zyklen) analog zu dem Schema bei Bonner et al. [34] gegeben wurde. Der experimentelle Arm für Kohorte 1 und 2 war eine chemotherapiefreie Kombination aus Cetuximab (wie im Kontrollarm von Kohorte 2) kombiniert mit Avelumab 10 mg/kg alle 2 Wochen. Nach Ende der CRT wurde Avelumab für bis zu 1 Jahr adjuvant fortgeführt. Die Randomisation erfolgte je Kohorte 1:1, und die Stratifikation erfolgte nach Behandlungszentrum, Nodalstatus (N0, N1 vs. N2, N3) und p16-Status (Oropharynxkarzinome p16+ vs. Oropharynxkarzinome p16−, Nichtoropharynxkarzinome). Über eine PD-L1-Testung oder entsprechende Subgruppenanalysen wurde bisher nichts berichtet.

Primärer Endpunkt war ebenfalls eine Verbesserung des PFS (Kohorte 1: HR $\leq$ 0,64; α‑Fehler mit zweiseitiger Signifikanz von 0,05 und Power = 80 %; Kohorte 2: HR $\leq$ 0,62; α‑Fehler mit einseitiger Signifikanz von 0,05 und Power = 80 %).

Von 09/2017 bis 08/2018 erfolgte eine einjährige Sicherheitsphase, während der 82 Patienten behandelt und keine Sicherheitsbedenken festgestellt wurden [30]. Von 2017 bis 2020 wurden insgesamt 707 Patienten randomisiert einem Studienarm zugeteilt, davon *n* = 430 Patienten in Kohorte 1 und *n* = 277 Patienten in Kohorte 2 [17].

In beiden Kohorten wurde der primäre Endpunkt verfehlt. Dies wurde bei der Jahrestagung der European Society of Medical Oncology (ESMO) 2021 berichtet [17].

In Kohorte 1 lag die PFS-Rate nach einem Jahr im experimentellen Arm bei 64 % (95%-KI: 54–72 %) und im Standardarm bei 73 % (95%-KI: 65–81 %). Dies entspricht einer HR von 1,27 (95%-KI: 0,83–1,93) [17]. Es war damit in der Zwischenanalyse ein Abbruchkriterium erfüllt. In der finalen Analyse mit 50,8 Monaten medianem Follow-up war die PFS-Rate nach 4 Jahren mit 42,3 % im experimentellen Arm signifikant schlechter als im Standardarm mit 54,7 % (HR = 1,4; 95%-KI: 1,07–1,82; *p* = 0,013) [16].

In Kohorte 2 lag die PFS-Rate nach 2 Jahren im Avelumab-Cetuximab-Arm bei 44 % (95%-KI: 35–53 %) und im Standardarm bei 31 % (95%-KI: 23–40 %). Daraus ergab sich eine HR von 0,84 (95%-KI: 0,62–1,15; *p* = 0,14) [17]. In der aktualisierten, finalen Auswertung beim ESMO-Kongress 2024 wurde über eine HR von 0,80 (95%-KI: 0,60–1,06; *p* = 0,059) berichtet. Für das OS bestand kein signifikanter Unterschied (HR = 1,05; 95%-KI: 0,76–1,43; *p* = 0,77) [16].

Die Studie war in beiden Kohorten auch in der finalen Auswertung negativ.

### Studie KEYNOTE-412

Die KEYNOTE-412-Studie war eine doppelt verblindete, placebokontrollierte, randomisierte Phase-III-Studie [15, 43]. Eingeschlossen wurden neudiagnostizierte HNSCC von Mundhöhle, Pharynx und Larynx im Stadium III–IVb gemäß AJCC-Klassifikation, Version 8 (Mundhöhle, Hypopharynx, Larynx, p16−-Oropharynx- bzw. bei p16+-Oropharynxkarzinomen T4 oder N3, Stadium III, mit gutem Performance-Status, ECOG 0/1), die für eine nichtchirurgische platinhaltige CRT mit Hochdosis-Cisplatin geeignet waren.

Die CRT konnte entweder mit 70 Gy normofraktioniert und 3‑mal Cisplatin 100 mg/m^2^ analog zur RTOG-91/11-Studie [49] oder akzeleriert (70 Gy in 6 Wochen mit 6 Fraktionen pro Woche) mit 100 mg/m^2^ analog zur RTOG-0129-Studie [41] durchgeführt werden. Im experimentellen Arm wurde Pembrolizumab 200 mg i.v. mit Beginn eine Woche vor Beginn der CRT alle 3 Wochen und adjuvant bis 12 Monate nach Ende der CRT gegeben. Im Standardarm erhielten die Patienten ein entsprechendes Placebo.

Die Randomisation erfolgte 1:1 und wurde stratifiziert nach der Radiotherapiefraktionierung (normofraktioniert vs. akzeleriert), Primärtumorregion und dem p16-Status (Oropharynxkarzinome p16+ vs. Oropharynxkarzinome p16−, Nichtoropharynxkarzinome) und Stadium (III vs. IV). Die PD-L1-Expression wurde mittels DAKO PharmDx Assay (22C3) der Fa. DAKO, (Carpinteria/CA, USA) bestimmt und mittels CPS quantifiziert. Der primäre Endpunkt war das ereignisfreie Überleben („event-free survival“, EFS). Das EFS war definiert als die Zeit von Randomisation bis zu einem definierten Ereignis. Als Ereignisse wurden Progression/Rezidiv in der Bildgebung (gemäß RECIST 1.1), ein histologischer Nachweis von invasivem Karzinom in der Primärtumorregion > 12 Wochen nach Ende der CRT, einer Salvage-Chirurgie am Primärtumor (falls darin invasives Karzinom nachgewiesen wurde), einer Salvage-Neck-Dissection > 20 Wochen nach Ende der CRT (falls invasives Karzinom darin nachgewiesen wurde) und Tod definiert. Für die Fallzahlstatistik wurde eine HR < 0,7 mit einem einseitigen α‑Fehler von 0,025 und Power = 94,6 % angenommen. Es wurden geplante Subgruppenanalysen definiert.

Insgesamt wurden 804 Patienten randomisiert einem Studienarm zugeteilt (je *n* = 402 in den Standard- und experimentellen Arm). Das Merkmal PD-L1-Expression war zwischen dem experimentellen und dem Standardarm relativ gleich verteilt. Von den 802 Patienten hatten 82 (10,1 %) einen CPS < 1 und 685 (85,2 %) einen CPS ≥ 1. Einen CPS ≥ 20 wiesen 291 Patienten auf (36,2 %).

Auch in der KEYNOTE-412-Studie wurde der primäre Endpunkt nicht erreicht. Die HR für das EFS lag bei 0,83 (95%-KI: 0,68–1,03; *p* = 0,043) in der Gesamtkohorte und in der Kohorte mit CPS ≥ 1 bei 0,8 (95%-KI: 0,64–1,00) [43]. Beim ESMO-Kongress 2022 wurden auch weitere Subgruppenanalysen präsentiert [14]. In der Subgruppe mit CPS ≥ 20 lag die HR für EFS bei 0,73 (95%-KI: 0,49–1,06).

### CRT + Immuntherapie – keine guten Partner?

Trotz der präklinischen Hinweise für eine Wirksamkeit der Kombination aus RT und Hemmung der PD1/PD-L1-Achse [31, 32] waren alle 3 Studien negativ. Die Ergebnisse der genannten 3 großen randomisierten Phase-III-Studien führten zu einer deutlichen Ernüchterung. Die Studien ähneln sich bezüglich der Einschlusskriterien, der Behandlungskonzepte und der Kontrollarme sehr stark, es gibt aber auch kleine Unterschiede.

Während die Studien JAVELIN Head and Neck 100 und die KEYNOTE-412 placebokontrollierte doppelt verblindete Studien waren, war die GORTEC-REACH-Studie unverblindet. Die zentralen Patientencharakteristika der Studien mit platinbasierter CRT als Standardarm sind in Tab. 3 dargestellt. Es bestehen signifikante Unterschiede bezüglich der Stadienverteilung, des p16-Status und der Primärtumorregion, aber nicht bezüglich des ECOG.

**Tab. 3:** Patientencharakteristika der Studien JAVELIN Head and Neck 100 [27], GORTEC-REACH (Kohorte 1) [16] und KEYNOTE-412 [43]

		JAVELIN Head and Neck 100	GORTEC-REACH-Kohorte 1	KEYNOTE-412	χ2	*p*-Wert
Kohortengröße (absolut)		697	426	804	n. a.	–
Stadium	Stadium III	58,4 %	18,3 %	34,3 %	192,4	< 0,0001
	Stadium IV	41,6 %	81,5 %	65,5 %		
p16	p16+	34,1 %	34,3 %	26,5 %	13	0,0015
	p16−	65,9 %	65,7 %	73,5 %		
ECOG	0	58,4 %	60,3 %	64,2 %	5,45	0,0654
	1	41,6 %	39,7 %	35,8 %		
Primarius	Mundhöhle	13,8 %	4,9 %	9,7 %	87,53	< 0,0001
	Oropharynx	46,8 %	70,2 %	50,2 %		
	Hypopharynx	21,7 %	17,1 %	17,9 %		
	Larynx	17,8 %	7,7 %	22,1 %		

*ECOG *Performance-Status der Eastern Cooperative Oncology Group

Die Ergebnisse der Studie JAVELIN Head and Neck 100 und der GORTEC-REACH-Studie ähneln sich deutlich. Beide Studien wurden nach einer geplanten Zwischenanalyse beendet und ergaben anstatt eines Vorteils numerisch eher einen Nachteil im PFS und OS. In beiden Studien gab es keine geplanten Subgruppenanalysen nach PD-L1-Expression. Während in einer explorativen Analyse der Studie JAVELIN Head and Neck 100 ein statistischer Trend zu einem Vorteil für PD-L1-positive Patienten beschrieben wurde, wurden von der GORTEC-REACH-Studie derartige Analysen nicht publiziert.

Einzig in der KEYNOTE-412-Studie gab es numerisch weder in der Gesamtkohorte noch für Patienten mit CPS ≥ 1 oder CPS ≥ 20 einen Hinweis für einen PFS- oder OS-Nachteil der Pembrolizumab-behandelten Patienten. Stattdessen lag in den explorativen Analysen ein numerischer EFS-Vorteil für Patienten mit CPS ≥ 1 oder CPS ≥ 20 vor. Im Gegensatz zur KEYNOTE-048-Studie, wo der hierarchische Analyseplan bei CPS ≥ 20 aus begann, wurde in der KEYNOTE-412-Studie die Analyse für die Gesamtkohorte begonnen. Dieser strategische Unterschied hätte gerade für Patienten mit CPS ≥ 20 die Auswertung verändern können. Eine weitere Frage ist, ob die Ergebnisse positiver gewesen wären, wenn eine PD-L1-Positivität (CPS ≥ 1 oder CPS ≥ 20) als Einschlusskriterium definiert worden wäre.

Inhaltlich werden in der Community verschiedene Ursachen diskutiert. Einerseits könnte die Standarddosis der RT (70 Gy für Primärtumor und betroffene Lymphknoten) die durch Hemmung der PD1/PD-L1-Achse expandierten, im Tumormikromilieu befindlichen tumorspezifischen Immunzellen [37] geschädigt haben, sodass die immunvermittelte Antitumoraktivität sich nicht entfalten konnte [13, 25, 44, 50]. In Konsequenz werden nun Therapiekonzepte diskutiert, die insbesondere die drainierenden Lymphknoten aussparen oder zumindest mit reduzierter Radiotherapiedosis behandeln [23].

Eine andere Hypothese resultiert aus Ergebnissen einer Phase-II Studie, in der eine kombinierte CRT kombiniert mit Pembrolizumab mit einem sequenziellen Ansatz mit adjuvantem Pembrolizumab erst nach Ende der CRT verglichen wurde [10]. In dieser Studie gab es einen numerischen Vorteil für die lokoregionäre Kontrolle in der sequenziell, also nur adjuvant behandelten Kohorte. Ähnliche Ergebnisse gab es beim nichtkleinzelligen Lungenkarzinom in der PACIFIC-Studie [8].

Eine vergleichbare Studie bei lokoregionär fortgeschrittenem HNSCC war die IMVOKE-010-Studie (NCT03452137), in der Atezolizumab adjuvant nach CRT eingesetzt wurde. In diese Studie konnten jedoch auch primär chirurgisch behandelte Patienten eingeschlossen werden. Die Studie wurde ebenfalls terminiert und konnte den primären Endpunkt (EFS) nicht erreichen [7].

Die aktuell noch rekrutierende, verblindete randomisierte Phase-III-Studie JADE untersucht derzeit den PD1-Antikörper Dostarlimab im Vergleich zu Placebo als adjuvante Therapie nach definitiver CRT mit dem primären Endpunkt EFS [6].

Eine weitere rekrutierende randomisierte Phase-III-Studie ist die eVOLVE-HNSCC-Studie, in der der bispezifische Antikörper Volrustomig (PD1 und CTLA4) adjuvant nach definitiver CRT im Vergleich mit Observation als Standardarm verglichen wird (unverblindet) [2].

## Neoadjuvante Therapie

Die neoadjuvanten Therapien waren Thema im Referat von Dr. Kürten und Dr. Ferris im Jahr 2024 [45]. In diesem Artikel wurden präklinische und bisher vorliegende klinische Daten ausführlich dargestellt.

Die KEYNOTE-689-Studie [18, 22] ist eine randomisierte Phase-III-Studie, in der im experimentellen Arm Pembrolizumab 200 mg alle 3 Wochen für 2 Zyklen neoadjuvant vor geplanter Chirurgie für resektable HNSCC von Mundhöhle, Pharynx und Larynx eingesetzt wird. Postoperativ wird Pembrolizumab während der adjuvanten (C)RT und danach als Monotherapie für insgesamt 1 Jahr appliziert. Vergleichsarm ist die gleiche Therapie ohne Pembrolizumab. In einer Pressemitteilung wurde am 08.10.2024 publiziert, dass die Studie ihren primären Endpunkt (EFS und „major pathologic response“) erreicht hat. Details der Studienergebnisse werden auf einem internationalen Kongress 2025 erwartet. Es ist davon auszugehen, dass auf Basis der Ergebnisse Pembrolizumab als neoadjuvante und adjuvante Therapie für resektable HNSCC zugelassen werden könnte. Genauere Ergebnisse sind bisher noch nicht bekannt, sodass noch nicht klar ist, ob die Zulassung nur für PD-L1-positive HNSCC oder für alle resektablen Patienten im Stadium III und IVa erfolgen wird.

Falls eine derartige Zulassung für die neoadjuvante Immuntherapie erfolgt, wird diese die Standardtherapie erheblich verändern und zu neuen Herausforderungen für die Behandler führen. Es erscheint daher ratsam, sich frühzeitig mit der medikamentösen Tumortherapie zu beschäftigen und Strukturen zu planen, die eine neoadjuvante Therapie auch in der Breite auf hohem Niveau möglich machen. Diese Strukturen sollten eine enge Einbindung der Kopf-Hals-Chirurgie in die Therapieplanung und Durchführung berücksichtigen.

Denn es gilt vor der neoadjuvanten Phase zu beachten, dass die Operation möglichst schon zu Beginn der Therapie terminiert und die Tumorausdehnung detailliert dokumentiert wird (Bildgebung, Fotos, Zeichnungen), um eine anatomische Resektion mit Sicherheitsabständen zu den alten Grenzen zu gewährleisten. Dies ist insbesondere notwendig, falls es zu einem Ansprechen kommt und die Tumorgröße rückläufig ist oder der Tumor gar nicht mehr erkennbar oder tastbar ist. Denn eine Resektion in alten Grenzen war in der KEYNOTE-689-Studie im Protokoll vorgesehen.

Tritt während der neoadjuvanten Therapie hingegen eine Progression ein, sollte die Resektabilität des Tumors erneut beurteilt werden, die geplante Resektion vorgezogen und die chirurgische Therapie und Rekonstruktion entsprechend adaptiert werden. Zudem ist es notwendig, immunvermittelte unerwünschte Arzneimittelwirkungen früh zu erkennen und korrekt zu behandeln.

Für die chirurgischen Fachgesellschaften, die Kopf-Hals-Tumoren behandeln, sind weitreichende Veränderungen der aktuellen Therapieabläufe erforderlich, um eine neoadjuvante Immuntherapie mit guten Ergebnissen zu gewährleisten.

## Fazit für die Praxis

- Kenntnisse in der medikamentösen Tumortherapie sind essenziell in der Behandlung von Plattenepithelkarzinomen im Kopf-Hals-Bereich (HNSCC).
- Immuntherapien, insbesondere PD1-Antikörper, sind in der palliativen Tumortherapie von HNSCC von zentraler Bedeutung in der Erst- und Zweitlinie.
- Platinhaltige Therapien und Cetuximab sind weiterhin relevant.
- Die Therapieauswahl sollte nach klinischen Kriterien (Combined Positive Score [CPS], Tumorsymptomlast, Progressionsdynamik, Vortherapie) personalisiert werden.
- In der kombinierten Chemoradiotherapie bleibt der Standard ein platinhaltige Therapie.
- Eine Zulassung für Pembrolizumab als neoadjuvante und adjuvante Therapie von resektablen HNSCC im Stadium III/IVa ist zeitnah wahrscheinlich.
- Strukturelle Änderungen sind erforderlich, um neoadjuvante Therapien optimal umzusetzen.

## Supplementary Information

Tabelle S1
