# Supplementary material for: Medical tumor therapy for head and neck cancer: between standardization and personalization
Source: HNO. 2025 Apr 22;74(2):97–109. [Article in German] doi: 10.1007/s00106-025-01591-7 (PMC12855352; doi:10.1007/s00106-025-01591-7)
Supplement: Supplementary file 1 — Tabelle S1 [file 106_2025_1591_MOESM1_ESM.pdf]

Supplementary Information

Tabelle S 1

Akronym	Indikation	Phase	Rando-misation	Experimenteller Arm	Kontrollarm	Beginn	Primäres Studienende	Ergebnis bzgl. primärem Endpunkt	Referenz
EXTREME	R/M HNSCC, Erstlinie, Platin-naïv	3	ja	Cetuximab, PF	PF	XX.12.2004	XX.03.2007	längeres OS. Zulassung Cetuximab + PF	52
TPExtreme	R/M HNSCC, Erstlinie, Platin-naïv	2	ja	Cetuximab TP	Cetuximab PF	10.10.2014	31.12.2021	negativ	26
CET MET	R/M HNSCC, Erstlinie, Platin-naïv	2	ja	Cetuximab Paclitaxel, Carboplatin	Cetuximab PF	01.11.2011	19.12.2017	non-inferiority (PFS, OS)	51

Checkmate-141	R/M HNSCC, Platin-refraktär	3	ja	Nivolumab	Prüferauswahl (T, MTX, Cetuximab)	29.05.2014	06.11.2015	längeres OS. Zulassung Nivolumab (Platin-refraktär)	11
Keynote-040	R/M HNSCC, Platin-refraktär	3	ja	Pembrolizumab	Prüferauswahl (T, MTX, Cetuximab)	17.11.2014	15.05.2017	längeres OS. Zulassung Pembrolizumab (Platin-refraktär TPS ≥ 50%)	12
EAGLE	R/M HNSCC, Platin-refraktär	3	ja	A) Durvalumab B) Durvalumab + Tremelimumab	Prüferauswahl (T, Paclitaxel, MTX, Cetuximab, F, Capecitabine, TS1)	09.09.2015	10.09.2018	negativ	38
Keynote-048	R/M HNSCC, Erstlinie, Platin-naïv	3	ja	A) Pembrolizumab B) Pembrolizumab + PF	Cetuximab PF	19.03.2015	25.02.2019	längeres OS. Zulassung Pembrolizumab, Pembrolizumab + PF (Erstlinie, CPS ≥ 1)	48

Checkmate-651	R/M HNSCC, Erstlinie, Platin-naïv	3	ja	Nivolumab + Ipilimumab	Cetuximab PF	05.10.2016	10.05.2021	negativ	29
KESTREL	R/M HNSCC, Erstlinie, Platin-naïv	3	ja	A) Durvalumab B) Durvalumab + Tremelimumab	Cetuximab PF	15.10.2015	06.07.2020	negativ	42
JAVELIN HN100	LAHNSCC, def. CRT	3	ja	Avelumab + CRT	CRT	28.11.2016	23.12.2019	negativ	27
GORTEC REACH	LAHNSCC, def. (C)RT	3	ja	A) Avelumab + CRT B) Avelumab + Cetuximab RT	A) CRT B) Cetuximab RT	14.09.2017	n.a.	negativ	16, 17
Keynote-412	LAHNSCC, def. (C)RT	3	ja	Pembrolizumab + CRT	CRT	05.04.2017	31.05.2022	negativ	15, 43

Keynote-689	LAHNSCC, neoadjuvant	3	ja	neoadjuvant / adjuvant Pembrolizumab + Chirurgie + Adjuvanz	Chiurgie + Adjuvanz	17.12.2018	25.07.2024	verlängertes EFS	press release
